# Discrimination and health: A cross-sectional study comparing Muslims with other-religious

**DOI:** 10.1177/14034948231225561

**Published:** 2024-03-22

**Authors:** Bushra Ishaq, Esperanza Diaz, Lars Østby

**Affiliations:** 1 Centre for Medical Ethics, Institute of Health and Society, Faculty of Medicine, University of Oslo, Norway; 2 MF Norwegian School of Theology, Religion and Society, Norway; 3 Department of Global Public Health and Primary Care, University of Bergen, Norway; 4 Statistics Norway, Oslo-Kongsvinger, Norway

**Keywords:** Perceived discrimination, Muslims, Norway, immigrants, self-rated health, mental health, Islam, social conditions, health outcome assessments

## Abstract

**Aims:**

The aim of this study is to report perceived discrimination among Muslims living in Norway and to address and compare associations between perceived discrimination and health among Muslims with an immigrant background and other-religious with an immigrant background.

**Method:**

A representative sample of individuals with an immigrant background in Norway was used in a cross-sectional study design that included 5484 respondents aged 16 to 74 years. The respondents were sub-grouped after religious affiliation, and as immigrants and Norwegian-born. This sample is from ‘The Survey on living conditions among persons with an immigrant background 2016’, conducted by Statistics Norway. Multivariate logistic regression analyses were conducted to investigate the relationship between perceived discrimination and self-rated health and between perceived discrimination and mental health problems.

**Results:**

Our findings show that Muslims with an immigrant background are more likely to report perceived discrimination than non-Muslims with an immigrant background. Perceived discrimination was associated with poor self-rated health and mental health problems among immigrant Muslims and Norwegian-born Muslims. Among other-religious with an immigrant background, perceived discrimination had an inverse relationship with mental health problems among immigrants, while an association between perceived discrimination and poor self-rated health was found among Norwegian-born.

**Conclusions:**

**Our findings suggest that perceived discrimination does play a role in health among minorities with an immigrant background in Norway, regardless of religion. However, the association between perceived discrimination and poor health seems to be stronger among Muslims, especially Norwegian-born Muslims.**

## Background

Discrimination is a determinant of poor health and is associated with negative health outcomes such as mental health problems, low self-rated health, obesity, low birth weight, negative healthcare behaviour and even mortality [1–6]. There are different pathways by which discrimination may act upon health. Racism is a trauma stressor that impacts the autonomic nerve system, the allostatic balance, the metabolic system, the immune system and the cardiovascular system [3,6,7]. Discrimination also has an effect on participation in the labour market, housing market and society in general, which may affect health through socioeconomic pathways [8,9]. Moreover, lack of trust, social exclusion and barriers to access to healthcare seem to play a role in how discrimination impacts health [3,5,10]. Discrimination is embedded in behaviour and is defined as unequal treatment of individuals or groups of individuals due to specific characteristics of the targeted group. The consequent disadvantages of such treatment are often included in the definition of racism [11]. There are causes of discrimination other than religion, and religious beyond Muslims do experience discrimination. Islamophobia is the subtype of racism that has increased most significantly globally, given the rise of the racialisation of Muslims [12,13]. Islamophobia targets not only Muslims but also individuals perceived as Muslims. Phenotype, expressed in features such as skin colour, is still an important cause of discrimination and may interplay or reinforce religion as a cause of discrimination [10]. The framework of intersectionality postulates that different social identities simultaneously and mutually intersect with structural factors in producing health disparities through disadvantages such as discrimination [14,15].

According to Statistics Norway, 60–70% of immigrants from Muslim countries have reported perceived discrimination [8,16]. Muslims are the minority towards which the majority population in Norway has most negative attitudes, the minority that experiences more social distance than other minorities, and is overrepresented as victims of hate crime in the Norwegian capital [9,17,18]. Perceived discrimination is a recurrent experience among Muslims that has increased across European countries [10,13]. Despite these facts, there has not been an adequate focus on Islamophobia within health research [4,19]. To our knowledge, no previous studies have described the association between perceived discrimination and health in a nationally representative sample of Muslims, including more than two nationalities and Western-born Muslims. This study aims to fill this gap.

## Aims

Our null hypotheses are: 1) Muslims are not more likely to report perceived discrimination than non-Muslims. 2) The association between perceived discrimination and health among Muslims is the same as the association between perceived discrimination and health among other-religious. 3) The association between perceived discrimination and health may depend on nativity, gender and religiosity. We included these effect modifiers as religiosity may function as a coping mechanism [4,20]. According to the framework of intersectionality, gender is a powerful concept involved in the intersection between macro-level systems of privileges (i.e. racism and sexism) and individual-level factors in producing health inequalities [14]. Nativity may represent differences in resources and societal exposure modifying the relationship between perceived discrimination and health.

## Method

### Sample and data collection

A cross-sectional study design was used. A national survey on living conditions among immigrants in Norway and their descendants is conducted recurrently by Statistics Norway. The survey aligns with the European Union Statistics on Income and Living Conditions. This study used the latest special survey (2016) dataset of immigrants and Norwegian-born individuals with immigrant parents.

This study did not require ethical approval from the Norwegian Regional Committee for Medical and Health Research Ethics, as the dataset was strictly anonymised. However, clearance was obtained from the Norwegian Centre for Research Data.

Individuals aged 16–74 years with backgrounds from 12 different countries of origin who had lived in Norway for at least two years were included using a probability sample from the Central Population Register. Statistics Norway defined immigrants in the survey as respondents born abroad with two foreign-born parents and Norwegian-born with an immigrant background as descendants of immigrant couples born in Norway. The response rate was 54.3%, and the total number of participants was 5484. The respondents could speak with an interviewer who spoke their mother tongue. A report from Statistics Norway provides further details about the data collection and the sample [21].

### Study groups

Muslims (*N*=2661; Table I) were defined from the sample according to the following criteria: 1) individuals reported to have been raised as Muslims and 2) individuals who identified themselves as Muslims when the interviews were conducted. Individuals who did not identify as Muslim, but were raised as Muslim, were not included in the sample as this group lacked Muslim religiosity as an exposure. (This group seems to differ from Muslims in relation between perceived discrimination and health. Supplemental material Tables III and V online show no association between perceived discrimination with self-rated health (SRH) and mental health problems in this group.)

**Table I.** Descriptive statistics of the samples.

	Immigrant Muslims	Immigrant other-religious	Norwegian-born Muslims	Norwegian-born other-religious
**Age**				
16–24 years	373 (17%)	102 (8%)	237 (50%)	273 (78%)
25–44 years	1154 (53%)	748 (54%)	233 (50%)	79 (22%)
45–74 years	664 (30%)	530 (38%)	0 (0%)	0 (0%)
Total	2191 (100%)	1380 (100%)	470 (100%)	352 (100%)
**Gender**				
Male	1188 (54%)	746 (54%)	251 (53%)	156 (44%)
Female	1003 (46%)	634 (46%)	219 (47%)	196 (56%)
Total	2191 (100%)	1380 (100%)	470 (100%)	352 (100%)
**Education**				
No education	80 (4%)	23 (2%)	1 (0 %)	0 (0%)
Primary and lower secondary school	945 (43%)	470 (34%)	195 (42%)	142 (40%)
Upper secondary school	514 (23%)	385 (28%)	119 (25%)	87 (25%)
University/university college	376 (17%)	302 (22%)	113 (24%)	76 (22%)
Missing	276 (13%)	200 (14%)	42 (9%)	47 (13%)
Total	2191 (100%)	1380 (100%)	470 (100%)	352 (100%)
**Employment**				
Not employed	952 (43%)	430 (31%)	148 (31%)	152 (43%)
Employed	1239 (57%)	950 (69%)	322 (69%)	200 (57%)
Total	2191 (100%)	1380 (100%)	470 (100%)	352 (100%)
**Self-reported financial status**				
Very difficult or difficult	566 (26%)	310 (22%)	35 (7%)	30 (9%)
Neither difficult or easy	1084 (50%)	705 (51%)	234 (51%)	169 (48%)
Quite easy or very easy	489 (22%)	338 (25%)	152 (32%)	75 (21%)
Missing	52 (2%)	27 (2%)	49 (10%)	78 (22%)
Total	2191 (100%)	1380 (100%)	470 (100%)	352 (100%)
**Importance of religion**				
No importance or little importance	148 (7%)	99 (7%)	8 (2%)	34 (10%)
Some importance	589 (27%)	453 (33%)	101 (21%)	188 (53%)
High or very high importance	1418 (65%)	817 (59%)	356 (76%)	130 (37%)
Missing	36 (1%)	11 (1%)	5 (1%)	0 (0%)
Total	2191 (100%)	1380 (100%)	470 (100%)	352 (100%)
**Religious attendance**				
No religious attendance	850 (39%)	207 (15%)	74 (16%)	50 (14%)
At least sometimes (seldom to several times a week)	1341 (61%)	1173 (85%)	396 (84%)	302 (86%)
Total	2191 (100%)	1380 (100%)	470 (100%)	352 (100%)
**Perceived discrimination**				
Never	1260 (57%)	903 (65%)	201 (43%)	176 (50%)
At least once	931 (43%)	477 (35%)	269 (57%)	176 (50%)
Total	2191 (100%)	1380 (100%)	470 (100%)	352 (100%)

Muslims were further categorised by place of birth into two subgroups: immigrant Muslims and Norwegian-born Muslims. The other study group is other-religious, that is, individuals who reported affiliation with a religion other than Islam (*N*=1732). A subdivision by place of birth was also applied for this group. Non-Muslims are considered as respondents who did not report affiliation to Islam.

### Variables

#### Main outcomes

We used two health outcomes as response variables: 1) SRH was measured by asking respondents to classify their health as very good, good, neither good nor poor, poor, or very poor [22]. Respondents who chose options ‘very good’ and ‘good’ were considered to have good health. 2) Mental health was measured using the five-item Hopkins Symptoms Checklist Scale and mental health problems were defined as a mean score of over 2 [23].

Perceived discrimination is the explanatory variable in this study. Respondents were asked whether they had experienced being treated differently in the 12 months preceding the interview 1) in the workplace, 2) in educational institutions, 3) in the healthcare system, 4) when applying for work, or 5) in any other situation. The last was followed by questions asking whether the respondents experienced discrimination 6) in any public office; 7) in contact with the police; 8) in the housing market; 9) at restaurants, coffee bars or nightclubs; 10) at stores or in banks; 11) on a bus, train or underground; 12) on the street or in squares. Given that not all questions were relevant to the respondents, those who answered ‘yes’ to one of these options were considered to have experienced perceived discrimination.

#### Analyses

SPSS version 26 was used to conduct the analyses.

We applied a chi-square test to compare reported perceived discrimination among Muslims and non-Muslims and relative risk as effect size. Assumptions for different models were checked prior to model selection. Logistic regression analyses were conducted to assess how perceived discrimination was associated with SRH and mental health problems. Directed acyclic graph was used to identify confounders, mediators and colliders. The analyses were adjusted only for confounders as we aimed to estimate the total exposure-outcome association. Age, gender, religiosity (measured by the importance of religion and religious attendance), education, employment and self-reported financial status were the identified confounders.

Supplemental Tables I and II present analysis using an interval scale variable for perceived discrimination. In evaluating gender, nativity and religiosity as moderators, multiple regression analyses were performed. Perceived discrimination and the interaction of perceived discrimination with gender, nativity and religiosity were included as independent variables. Overall, including the dataset with immigrants and the dataset with Norwegian-born individuals with an immigrant background, the total amount of missing data was 1.6%. However, the variable of the self-reported financial situation gave high missing value as several Norwegian-born individuals were young and the variable did not apply to them. Hence, imputation was conducted for both datasets using multiple imputation methods.

## Results

Table I describes the characteristics of the study groups. Muslims were 4% more likely to report perceived discrimination than non-Muslims (Table II). The chi-square test was significant. The relative risk was 1.10 (95% confidence interval 1.03–1.17), implying a 10% increased risk of perceived discrimination among Muslims. The in-group difference was, however, more considerable. Norwegian-born Muslims were 13% more likely to report perceived discrimination than immigrant Muslims.

**Table II.** Perceived discrimination by affiliation to Islam and place of birth among Muslims.

**PD by affiliation to Islam**				
	Non-Muslims	Muslims	*p*	Relative risk
No PD	1659 (59%)	1461 (55%)		
PD	1160 (41%)	1200(45%)	**	1.10 (1.03–1.17)
Total	2819 (100%)	2661 (100%)		
**PD by place of birth among Muslims**				
	Immigrant Muslims	Norwegian-born Muslims	*p*	Relative risk
No PD	1260 (57%)	201 (44%)		
PD	931 (43%)	269** (56%)	**	1.35 (1.20–1.50)
Total	2191 (100%)	470 (100%)		

* *p* <0.05

** *p*<0.01.

PD: perceived discrimination

Among immigrant and Norwegian-born Muslims, perceived discrimination was associated negatively with good SRH (Table III) and positively with mental health problems in the adjusted model for all confounders (Table IV). Perceived discrimination was related to mental health problems in the same model among other-religious immigrants, but no association between perceived discrimination and SRH was found. Perceived discrimination had an inverse relationship with good SRH in the adjusted model for all confounders among Norwegian-born other-religious but showed no significant association with mental health problems.

**Table III.** Associations between perceived discrimination and good self-rated health.

	Model 1	Model 2	Model 3	Model 4
Immigrant Muslims	1.07 (0.89–1.30)	0.76** (0.62–0.92)	0.75** (0.61–0.91)	0.75** (0.61–0.93)
Immigrant other-religious	1.40* (1.10–1.74)	1.03 (0.78–1.34)	1.03 (0.80–1.35)	1.10 (0.83–1.45)
Norwegian-born Muslims	0.54* (0.32–0.93)	0.55* (0.32–0.95)	0.53* (0.31–0.92)	0.47* (0.26–0.83)
Norwegian-born other-religious	0.45* (0.22–0.89)	0.44* (0.20–0.89)	0.42* (0.21–0.85)	0.42* (0.21–0.86)

Odds ratio and 95% confidence interval. Model 1: unadjusted. Model 2: adjusted for age and gender. Model 3: adjusted for age, gender, religiosity. Model 4: adjusted for age, gender, religiosity, education, employment and financial situation.

* *p* value is significant at the 0.05 level (two-tailed).

** *p* value is significant at the 0.01 level (two-tailed).

**Table IV.** Associations between perceived discrimination and mental health problems.

	Model 1	Model 2	Model 3	Model 4
Immigrant Muslims	2.00** (1.60–2.40)	2.30** (1.82–2.83)	2.31** (1.84–2.88)	2.20** (1.74–2.80)
Immigrant other-religious	1.72** (1.30–2.34)	1.83** (1.33–2.52)	1.82** (1.32–2.50)	1.70** (1.20–2.31)
Norwegian-born Muslims	3.40** (1.70–7.10)	3.34** (1.63–6.90)	3.51** (1.70–7.32)	3.82** (1.77–8.22)
Norwegian-born other-religious	1.77 (0.86–3.64)	1.77 (0.86–3.64)	1.84 (0.89–3.81)	1.87 (0.90–3.89)

Odds ratio and 95% confidence interval. Model 1: unadjusted. Model 2: adjusted for age and gender. Model 3: adjusted for age, gender, religiosity. Model 4: adjusted for age, gender, religiosity, education, employment and financial situation

* *p* value is significant at the 0.05 level (two-tailed).

** *p* value is significant at the 0.01 level (two-tailed).

The same results were found in the analysis using an interval scale for perceived discrimination for all study groups (Supplemental Tables I and II).

In the interaction analysis, we did not find any significant association interaction effect with religiosity, nativity or gender.

## Discussion

Our findings indicate that perceived discrimination might be common among individuals with an immigrant background in Norway. However, the prevalence of perceived discrimination is higher among Muslims than non-Muslims. These findings and the in-group difference among Muslims are consistent with previous research on individuals from Muslim countries. However, these studies did not group their respondents by religious affiliation but by country of origin [8,10,16,17].

Perceived discrimination was associated with poor SRH and mental health problems among Muslims. The same applied, with an even stronger association, for Norwegian-born Muslims, although this group is better qualified to be integrated into society. Norwegian-born Muslims are overrepresented in higher education attainment in Norway [24]. Several studies have found a harmful effect of discrimination on health regardless of the social status or context of the experience [6,8]. Using an intersectional approach to explain these generational differences, nativity presents a relocation in socioeconomic status due to mobilisation in class among Muslims and a change in how the majority population perceives Muslim identity due to the post-9/11 increase of racialisation of Muslim [12,13,15,25]. Both changes may intersect in making Norwegian-born Muslims a more relevant target for discrimination than their parental generation, making them more capable of reporting discrimination and more vulnerable to the harmful consequences of perceived discrimination [14,15]. Simultaneously, this generation may have high expectations of acceptance, as Norwegian Muslims generally report a strong sense of belonging to Norway [25].

A strong sense of belonging seems not to be a sufficient buffer to ameliorate the effects of perceived discrimination. Previous studies show more adverse health consequences of perceived discrimination related to a stronger community belonging. Such advancement is discussed to be attributed to the importance of acceptance by the discriminating group [7,26].

As perceived discrimination is associated with less trust in the country of residence among Muslims, these factors may also be important in how perceived discrimination may affect health, especially regarding resources to cope with perceived discrimination [8,10,19]. The higher exposure to society with increased potential of perceived discrimination may also explain the generational differences [8]. We find support for this reasoning from analysis treating perceived discrimination on an interval scale (Supplemental Tables I and II). Accordingly, our contribution to the theoretical framework of intersectionality indicates the involvement of nativity and Muslim identity in a complex intersection with other disadvantaging identities in facilitating health disparities.

Perceived discrimination was associated with mental health problems among other-religious immigrants but not among Norwegian-born other-religious. This finding might have occurred because the latter group is young and limited in size. However, an association between perceived discrimination and SRH was evident among Norwegian-born other-religious and was consistent throughout generations of Muslims. Although some studies have described the association between perceived discrimination and mental health problems as more consistent than the association between perceived discrimination and poor physical health, there is growing evidence that perceived discrimination also predicts impaired physical health [1,19]. A British study assessed associations between perceived discrimination and health among Muslims and included control groups in a longitudinal design. This study found worse health outcomes due to perceived discrimination as measured by blood pressure, serum cholesterol, mental health and SRH [3]. Further, American studies have also found an association between Islamophobia and poor physical health [2,4]. A Finnish study that included individuals with an immigrant background (Somali, Russian and Kurdish origin) found that experiences of discrimination increased the odds of poor SRH, mental health problems and disability [19].

According to a meta-analysis, ethnicity moderates the effect of perceived discrimination on health.[6] The context of the experience of perceived discrimination, the level of discrimination, and available resources to cope with perceived discrimination play a role in how perceived discrimination impacts health. Consequently, not all possible associations between perceived discrimination and health exist in every targeted group of racism [4,5]. Previous research has found that immigrants from Muslim countries have fewer coping resources, including social capital, than immigrants from non-Muslim countries [8]. To some extent, this lack of resources may explain the different findings among our study groups.

Concerning our third hypothesis, although religion has been described as a coping mechanism, this explanation seems not to be the case in our study. Previous studies have found that religiosity relates to health-promoting factors, but these associations seem to depend on the surrounding society. In religious societies and societies where religion is highly appreciated, religiosity is more closely linked to positive health outcomes than is the case in secular societies [27].

Our findings are relevant for public health policies in a historical context 20 years after 9/11 and relevant to the current Black Lives Matter movement. As perceived discrimination seems related to poor health across different minorities, it is essential to question perceived discrimination as a determinant of poor health of minorities. Although our study did not elucidate an association between perceived discrimination and health among the majority population, perceived discrimination may also threaten the wellbeing of society as a whole. Measures against discrimination should be included in policies addressing health disparities among minorities. During the COVID-19 pandemic, such countermeasures were missing in Norway, especially among healthcare authorities [20].

As discrimination also occurs within the healthcare system, healthcare professionals and health authorities should take a clear stand against discrimination [8]. More research on the role of discrimination in health disparities is also needed, such as how perceived discrimination is related to objective measured health outcomes such as blood-pressure, the role of perceived discrimination in health behaviour and healthcare seeking among minorities. Also, the pathways for how perceived discrimination predicts health disparities should be addressed.

Compared with previous research, the strength of this study relates to the representative sample, comparison groups, and inclusion of religiosity as moderator and SRH as an outcome. This study is also unique because it has been conducted in Europe; most available studies on Islamophobia and health have been conducted in the USA. As Norway has a publicly funded healthcare system, this study has an advantage compared with similar American studies by reducing the interfering impact of formal barriers to healthcare access. However, the findings of this study are supported by American studies reporting a relationship between perceived discrimination with poor general health, mental health problems and poor physical health outcomes such as adverse birth outcomes [2,4,28,29]. Also, a more adverse relationship between perceived discrimination and health is described among American-borns with immigrant backgrounds compared with their parental generation [15].

To our knowledge, only one previous study has found an association between perceived discrimination and poor health among immigrants in Norway. This study found an association between perceived discrimination and mental health but no significant association between perceived discrimination and SRH [5]. In comparison, we found an association between perceived discrimination and SRH. This difference may be explained by our inclusion of Norwegian-born individuals, by our grouping of respondents by religious affiliation, and that Muslims report perceived discrimination more often than non-Muslims with an immigrant background.

Our study has several limitations. Given the cross-sectional design, we cannot make any conclusions on cause and effect in associations between perceived discrimination and health outcomes. A reverse causality is possible, as stigma related to illness, especially mental health disorders, can lead to discrimination. Poor health represents another disadvantaged identity that may intersect in a complexity causing health inequalities according to the framework of intersectionality. Further development of the hypothesis could include the role of pre-existing illness in discrimination and how such a pathway can exacerbate sickness.

However, previous research employing longitudinal designs has mostly found that perceived discrimination predicts poor health rather than the opposite [1,3].

Although the sample in this study is the latest national survey among immigrants from Statistics Norway, the data are from 2016, and we have experienced a pandemic in between. Accordingly, we reflect on the potential of conservative estimates of the relations investigated in this study. Other limitations are that the population of Norwegian-born individuals is relatively young, especially among other-religious; the questions used to measure discrimination have not been validated despite similarities with the validated 20-item Perceived Discrimination Scale; and perceived discrimination is a subjective parameter based on perception rather than reality [11]. Although our respondents report perceived discrimination, it is not given that they are objectively discriminated against. Individuals may also report an experience attributed to discrimination differently, and some may not even perceive it as perceived discrimination. However, an advantage of such a measurement is that it enables one to assess the extent of discrimination in large representative samples [8,11]. Moreover, many studies on discrimination use self-reported discrimination as a measurement [4,6]. Furthermore, both of our health outcome variables are validated measures [22,23]. As visible religious practices might be a driver for perceived discrimination, a limitation herein might be the numbers of available measures of individual religiosity as the only available measures for investigating the moderating role of religion were the importance of religion and religious attendance [30]. Finally, we report a risk of same-source bias using self-reported variables, especially when studying mental health as an outcome. Respondents’ mental health may have influenced the perception of discrimination.

## Conclusion

Perceived discrimination seems associated with poor physical and mental health problems among minorities with an immigrant background in Norway. However, these associations were stronger among Muslims, especially Norwegian-born Muslims. In addressing health disparities, discrimination should be questioned as a potential threat to the health of minorities with immigrant backgrounds.

## Supplemental Material

sj-docx-1-sjp-10.1177_14034948231225561 – Supplemental material for Discrimination and health: A cross-sectional study comparing Muslims with other-religious Supplemental material, sj-docx-1-sjp-10.1177_14034948231225561 for Discrimination and health: A cross-sectional study comparing Muslims with other-religious by Bushra Ishaq, Esperanza Diaz and Lars Østby in Scandinavian Journal of Public Health
