# Corrigendum

**DOI:** 10.1177/14034948251346412

**Published:** 2025-06-09

Ishaq B, Diaz E, Østby L. Discrimination and health: A cross-sectional study comparing Muslims with other-religious. *Scandinavian Journal of Public Health*. 2024;53(3):242-249. doi:10.1177/14034948231225561

In the above article, the title should have read:

Discrimination and health: A cross-sectional study comparing Muslims with other religious groups.

