# Supplementary material for: Discrimination and health: A cross-sectional study comparing Muslims with other-religious
Source: Scand J Public Health. 2024 Mar 22;53(3):242–9. doi: 10.1177/14034948231225561 (PMC12012278; doi:10.1177/14034948231225561)
Supplement: sj-docx-1-sjp-10.1177_14034948231225561 – Supplemental material for Discrimination and health: A cross-sectional study comparing Muslims with other-religious [file sj-docx-1-sjp-10.1177_14034948231225561.docx]

**Supplementary Tables article 2**

**Table 1: Associations between perceived discrimination and good self-rated health using an interval scale for PD as exposure.**^1,2^

|  | **Model 1: crude** | **Model 2: adjusted** |
| --- | --- | --- |
| **Immigrant Muslims** | 0.96 (0.88-1.04) | 0.82** (0.75-0.91) |
| **Immigrant other-religious** | 1.09 (0.95-1.25) | 0.97 (0.84-1.13) |
| **Norwegian-born Muslims** | 0.75** (0.63-0.89) | 0.70** (0.58-0.85) |
| **Norwegian-born other-religious** | 0.75*(0.58-0.98) | 0.75* (0.58-0.99) |

**^1^**Odds ratio and 95% confidence interval. Model 1: unadjusted. Model 2: adjusted for age, gender, religiosity, education, employment and financial situation. *P value is significant at the .05 level (2-tailed). **P value is significant at the .01 level (2-tailed).

**^2^**This variable was coded with values 0,1,2,3 and 4. Value 0 implied no episodes of PD in the preceding 12 months, while the three ascending values implied 1, 2, and 3 episodes of PD. As few respondents reported more than 4 episodes of PD, 4 or more episodes of PD were operationalized as the highest value in this scale.

**Table 2: Associations between perceived discrimination and mental health problems using an interval scale for PD as exposure.^1,2^**

|  | **Model 1: crude** | **Model 4: adjusted** |
| --- | --- | --- |
| **Immigrant Muslims** | 1.38**(1.26-1.50) | 1.45**(1.32-1.60) |
| **Immigrant other-religious** | 1.44**(1.25-1.66) | 1.42**(1.22-1.65) |
| **Norwegian-born Muslims** | 1.67**(1.37-2.03) | 1.89**(1.50-2.39) |
| **Norwegian-born other-religious** | 1.25 (0.95-1.66) | 1.25 (0.93-1.66) |

**^1^**Odds ratio and 95% confidence interval. Model 1: unadjusted. Model 2: adjusted for age, gender, religiosity, education, employment and financial situation. *P value is significant at the .05 level (2-tailed). **P value is significant at the .01 level (2-tailed)

**2**This variable was coded with values 0,1,2,3, and 4. Value 0 implied no episodes of PD in preceding 12 months, while the three ascending values implied respectively 1, 2, and 3 episodes of PD. As there were few respondents who reported more than 4 episodes of PD, 4 or more episodes of PD were operationalized as highest value in this scale.

**Table 3: Associations between perceived discrimination with good self-rated health and mental health problems among non-Muslims raised as Muslims (N=325). Exposure is a binary variable for PD.^1^**

|  | **Model 1: crude** | **Model 2: adjusted** |
| --- | --- | --- |
| **Self-rated health** | 1.13 (0.70-1.84) | 0.81 (0.45-1.45) |
| **Mental health problems** | 1.91* (1.12-3.30) | 1.90 (1.02-3.57) |

**^1^**Odds ratio and 95% confidence interval. Model 1: unadjusted. Model 2: adjusted for age, gender, religiosity, education, employment and financial situation. *P value is significant at the .05 level (2-tailed). **P value is significant at the .01 level (2-tailed)

**Table 4: Associations between perceived discrimination with good self-rated health and mental health problems among non-Muslims raised as Muslims (N=325). Exposure is interval scale- variable for PD.^1^**

|  | **Model 1: crude** | **Model 2: adjusted** |
| --- | --- | --- |
| **Self-rated health** | 1.16 (0.95-1.43) | 1.06 (0.83-1.39) |
| **Mental health problems** | 1.22 (1.00-1.48) | 1.26 (1.00-1.58) |

**^1^** Odds ratio and 95% confidence interval. Model 1: unadjusted. Model 2: adjusted for age, gender, religiosity, education, employment and financial situation. *P value is significant at the .05 level (2-tailed). **P value is significant at the .01 level (2-tailed)
